# Low Incidence of Avian Predation on the Brown Marmorated Stink Bug, *Halyomorpha halys* (Hemiptera: Pentatomidae), in Southeastern Orchard Systems

**DOI:** 10.3390/insects14070595

**Published:** 2023-07-01

**Authors:** Erin E. Grabarczyk, Ted E. Cottrell, Jason M. Schmidt, P. Glynn Tillman

**Affiliations:** 1 Southeast Watershed Research Laboratory, USDA-ARS, Tifton, GA 31793, USA; glynn.tillman@usda.gov; 2 Department of Biology, Valdosta State University, Valdosta, GA 31698, USA; 3 Southeastern Fruit and Tree Nut Research Laboratory, USDA-ARS, Byron, GA 31008, USA; ted.cottrell@usda.gov; 4 Department of Entomology, University of Georgia, Tifton, GA 31793, USA; jschmid2@uga.edu

**Keywords:** biological control, molecular gut content analysis, arthropod prey, pecan orchard, peach orchard

## Abstract

**Simple Summary:**

Stink bugs (Hemiptera: Pentatomidae) are an insect pest that cause damage to pecan and peach fruits. In forests that surround fruit and tree nut orchards, insectivorous birds may consume stink bug pests, such as the brown marmorated stink bug (*Halyomorpha halys*). In this study, we mist-netted birds, collected avian fecal samples, and monitored brown marmorated stink bugs in forests that surround peach and pecan orchards in Georgia, USA. We used PCR to screen avian fecal samples for brown marmorated stink bug DNA, which is evidence of their consumption by birds. We found three bird species consumed brown marmorated stink bugs, including Northern cardinal (*Cardinalis cardinalisis*), Tufted titmouse (*Baeolophus bicolor*), and Carolina wren (*Thryothorus ludovicianus*). Overall, the number of avian fecal samples with brown marmorated stink bug DNA was low, which may be due to the short retention time of prey for birds. Future studies should explore whether birds contribute to the biological control of additional pecan and pecan insect pests.

**Abstract:**

In many agroecosystems, brown marmorated stink bugs (*Halyomorpha halys*) (Hemiptera: Pentatomidae) are polyphagous pests that cause significant economic losses to numerous crops every year. Insectivorous birds may provide a means of sustainable predation of invasive pests, such as *H. halys*. In forest margins surrounding peach, pecan, and interplanted peach–pecan orchards, we monitored *H. halys* populations with pheromone-baited traps, mist-netted birds, and collected avian fecal samples for molecular gut content analysis. We screened 257 fecal samples from 19 bird species for the presence of *H. halys* DNA to determine whether birds provide the biological control of this pest. Overall, we found evidence that four birds from three species consumed *H. halys*, including Northern cardinal (*Cardinalis cardinalisis*), Tufted titmouse (*Baeolophus bicolor*), and Carolina wren (*Thryothorus ludovicianus*). *Halyomorpha halys* captured in traps increased over time but did not vary by orchard type. Although incidence of predation was low, this may be an underestimate as a result of our current avian fecal sampling methodology. Because birds are members of the broader food web, future studies are needed to understand avian ecosystem services, especially in terms of pest control, including *H. halys* and other pest species.

## 1. Introduction

Brown marmorated stink bugs (*Halyomorpha halys*) (Stål) (Hemiptera: Pentatomidae) are an invasive pest that aggregates in urban and agricultural areas, including orchards [1,2,3]. In orchard agroecosystems, *H. halys* disperse between fruit trees and surrounding unmanaged habitats, foraging on available foods, which results in significant economic damage [4,5,6,7,8,9]. Control of *H. halys* often relies on chemical spray applications targeted on trees within orchards [1,2,3]. As a result, management practices are usually applied within the boundaries of crop fields and orchards, while the surrounding non-crop habitats, such as forests, woodlands, and hedgerows, which can support populations of *H. halys*, are left untouched. Because *H. halys* disperse between agricultural and surrounding habitats, solutions for their management should seek strategies to include surrounding non-crop habitats.

Insectivorous birds that inhabit forest habitats surrounding orchards may provide biological control of arthropod pests [10,11]. Molecular gut content analysis of avian fecal material (i.e., gut content) is a minimally disruptive approach, where samples are easily collected during routine mist-netting procedures in and around agricultural areas [12]. To date, analysis of avian pest consumption ranges from detection of a single prey species [13,14] to DNA metabarcoding analysis that captures diet breadth [15,16,17]. Although more research is warranted, some general patterns of avian pest control in orchard systems have emerged. In macadamia orchards, an analysis of eleven bird species found overlaps in diet diversity and consumption of five major insect pest species, including pentatomids [15]. In this system, as pest populations increased in orchards, consumption of insect pests by birds increased as well [15]. A DNA-based approach was used to detect whether birds forage on codling moth (*Cydia pomonella*) (L.) (Lepidoptera: Tortricidae) in organic apple orchards, however, detection was extremely low; only one species, the brown-headed cowbird (*Molothrus ater*), ate codling moths [13]. To date, no research has focused specifically on birds as biocontrol agents of the brown marmorated stink bug, a significant agricultural pest that is rapidly expanding across the southeastern USA [18].

The objective of this study was to assess bird contributions to the biological control of the brown marmorated stink bug, *H. halys*, in forest habitats adjacent to commercial pecan and peach orchards. During 2021–2022, we monitored *H. halys* populations with pheromone-baited traps at nine orchards in central Georgia, USA. Simultaneously, we mist-netted birds and collected avian fecal samples for molecular gut content analysis. We used targeted PCR to determine the presence or absence of *H. halys* DNA in avian fecal samples and compared counts of *H. halys* captured in forests surrounding orchards to their consumption by birds.

## 2. Materials and Methods

### 2.1. Field Methods

**Figure 1:**
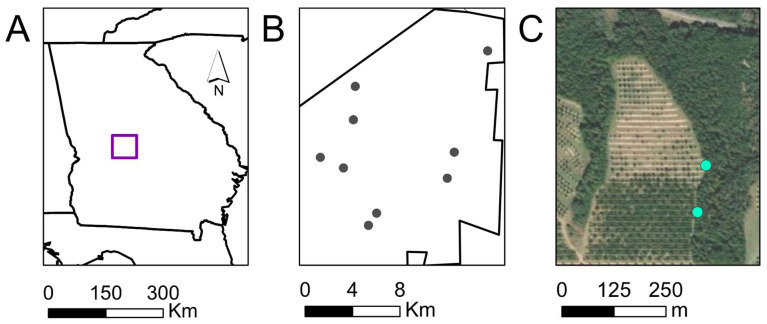
Map image showing location of study area marked with a purple square (**A**) in Peach County, Georgia, USA that included nine orchards (gray points) (**B**). At each site, two pheromone-baited traps (**C**) were placed in forest habitat surrounding orchards to monitor *Halyomorpha halys.* Birds were captured in mist nets and fecal samples collected in forest margins surrounding the orchards (turquoise points).

To capture and monitor *H. halys*, we used yellow pyramid traps, baited with aggregation pheromones highly attractive to nymphs and adults of *H. halys*, within approximately a 3 m^2^ area [19,20,21,22,23]. Two pheromone-baited traps were set along forest edge adjacent to peach and pecan orchards at nine study sites (*n* = 18 traps) from 1 June 2021 to 1 September 2021, and at seven of the same nine study sites (*n* = 14 traps) from 5 May 2022 to 6 July 2022. Three of the sites were pecan orchards, three were peach orchards, three were interplanted pecan and peach orchards, and all were located in Peach County, Georgia, USA (Figure 1A,B). Traps were placed at least 50 m apart in forests along the forest-orchard border (Figure 1C). Pheromone-baited traps consisted of a 2.8-L clear plastic PET jar with a screw-top lid (10.2-mm diameter) to collect insects (United States Plastic Corp., Lima, OH). Collection jars were placed on top of a 1.22 m yellow pyramid trap base [24]. The commercially available pheromone lures for *H. halys* includes the aggregation pheromone of *H. halys*, a combination of stereoisomers (3S,6S,7R,10S)-10,11-epoxy-1-bisabolen-3-ol and (3R,6S,7R,10S)-10,11-epoxy-1 bisabolen-3-ol (PHER), and a synergist, methyl (2E,4E,6Z)-2,4,6-decatrienoate (MDT), which is the aggregation pheromone of *Plautia stali* (Scott) (Hemiptera: Pentatomidae). Therefore, to increase capture success of *H. halys*, we baited traps with a set of two commercial aggregation lures: *H. halys* male aggregation pheromone (PHER) and the synergist (MDT) (Trécé Pherocon, Adair, OK, USA). Inside each trap, we placed a kill strip (10% λ-cyhalothrin and 13% piperonyl butoxide) (Saber extra insecticide ear tags, Sagebrush Tags, De Smet, SD) to decrease the likelihood of stink bug escape [25]. Once per week, captured stink bugs were removed from traps and stored in resealable bags at the Southeast Watershed Research Laboratory in Tifton, Georgia. Pheromone baits were replaced every other week. Identification of *H. halys* was based on Rice et al. [3]. Pesticide application (fungicide, insecticide, and herbicide) at study sites followed recommendations for commercial peach and pecan production published by the University of Georgia extension guidelines [26,27].

From 8 June 2021 to 26 August 2021 and from 5 May 2022 to 27 June 2022, birds that inhabited forests surrounding focal orchards were captured via mist net and fecal gut content samples were collected. Each morning of netting, we opened one to four 30 mm nylon mist nets (6 m and 9 m; Avinet Research Supplies, Portland, ME, USA) at dawn, and closed nets between 10:00 and 14:00, depending on ambient temperature. The site selected for netting and banding was determined with a random number generator (random.org) (accessed on 27 June 2022), however, if chemical applications were scheduled for a particular orchard block on the same day as netting, the next available site was sampled. The placement of nets within forest margins ranged from 1 m (at the edge of forest adjacent to orchard) to approximately 200 m from the forest–orchard border. Mist nets were placed in areas where birds were active and singing. During mist-netting, we broadcast species-specific playbacks from a Bluetooth speaker to lure individuals into nets (JBL Clip2; Harman International Industries, Stamford, CT, USA). Mist nets were checked every 10 min. We removed birds from the mist net, and captured individuals were transferred to paper holding bags. Each individual bird was kept for no more than 10 min. We banded birds with a United States Geological Survey (USGS) aluminum band and collected fecal samples either while handling the bird or extricated fecal material from holding bags with sterilized forceps. Fecal samples were placed in a 1.5 mL microcentrifuge tube that contained 0.5 mL RNALater solution (Thermo Fisher, Waltham, MA, USA). Samples were kept on ice until transferred to a −20 °C freezer upon return to the laboratory.

### 2.2. Molecular Gut Content Analysis and PCR Protocol

We used the DNeasy PowerSoil kit (Qiagen, Hilden, Germany) to extract arthropod DNA following the manufacturer’s instructions. For every 48 samples, a negative control was extracted, which did not contain avian fecal material. We included negative control extractions on plates during PCR preparation. In addition, we extracted DNA from the legs of an adult *H. halys* and included this extract on each plate as our positive control. We used 16S species-specific primers (16Sbr-H and 16Sar-L) to amplify *H. halys* present in avian fecal samples (as designed by the authors of reference [28]). PCRs were conducted at a scale of 12.5 µL containing 6.25 µL Qiagen Multiplex 2× Master mix (Qiagen, Hilden, Germany), 0.31 µL BSA, 0.625 µL of each primer (forward and reverse), 2.69 µL PCR grade H_2_O, and 3 µL of extracted avian fecal DNA. The thermocycling conditions for reagents were 95 °C for 15 min followed by 40 cycles of 94 °C for 45 s, 61 °C for 45 s, 72 °C for 30 s, and a final extension of 72 °C for 5 min. All PCR products were visualized on a Qiaxcel Advanced System (Qiagen, Hilden, Germany) for evaluation of positive incidence of predation on *H. halys* (threshold of >0.075 RFUs, [29], Supplemental Materials Table S1), in order to provide evidence of *H. halys* DNA in avian fecal samples.

### 2.3. Statistical Analysis

We tested whether the mean number of *H. halys* (adults and nymphs) varied according to orchard type, collection date, and year. First, we calculated the mean number of *H. halys* captured by site (2 traps per site) for each collection date. We fit a general linear mixed effect models in R version 4.2.2 program software (R CoreTeam 2022) using the package lme4 (function: lmer; [30]). For our model, we included orchard type (peach, pecan, and interplanted), collection date (centered and scaled), and collection year (2021 or 2022) as fixed effects, and site as a random effect. We log transformed the mean number of *H. halys* and used residual plots to assess model fit.

## 3. Results

### 3.1. Total H. halys and Birds Captured

**Figure 2:**
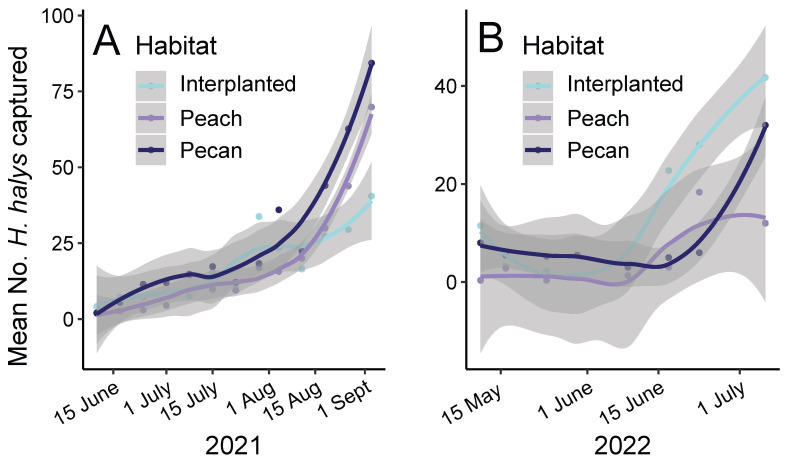
Mean number of *H. halys* captured in pheromone-baited traps by habitat in forests surrounding commercial orchards in Peach County, Georgia, USA during the years (**A**) 2021 and (**B**) 2022. A regression line (Loess) and 95% confidence intervals are displayed for weekly means by habitat (points).

A total of 5849 *H. halys* (adults and nymphs) were captured in pheromone-baited traps positioned in forest margins surrounding orchards during 2021–2022. *Halyomorpha halys* were trapped at all sites. We collected avian fecal samples from 257 individuals from 19 different bird species (Table 1). The six bird species captured most often included Carolina wren (*Thryothorus ludovicianus*), Eastern towhee (*Pipilo erythrophthalmus*), Field sparrow (*Spizella pusilla*), Northern cardinal (*Cardinalis cardinalisis*), Tufted titmouse (*Baeolophus bicolor*), and White-eyed vireo (*Vireo griseus*). The mean number of *H. halys* differed significantly according to collection date and year but not habitat (i.e., interplanted, peach, pecan; Figure 2). Specifically, the mean number of *H. halys* captured increased over the season (*F*_1, 158_ = 116.8, *p* < 0.0001; Figure 2) and, on average, more individuals were captured during 2021 (mean = 21.0 stink bugs per trap) than during 2022 (mean = 8.6 stink bugs per trap; *F*_1, 166_ = 8.5, *p* = 0.004; Figure 2).

### 3.2. Foraging Patterns of Birds

We detected *H. halys* consumption in 4 out of 257 avian fecal samples. Two Carolina wrens ate *H. halys* in forests surrounding peach orchards during August 2021. In interplanted peach and pecan orchards, one Tufted titmouse and one Northern cardinal ate *H. halys* during July 2021 and May 2022, respectively. We found no evidence of *H. halys* consumption by birds in forests that surround pecan orchards.

## 4. Discussion

For insectivorous birds, retention time of prey may occur over a short period of time, approximately 30 min to 4 h post-feeding [31,32,33]. Therefore, our results likely underestimate avian predation on *H. halys* based on our sample design and methodology. Nevertheless, we found three bird species consumed *H. halys* in forests surrounding orchards: Northern cardinal, Tufted titmouse, and Carolina wren. Both Northern cardinals and Tufted titmice are omnivorous and consume a mixed diet of seeds, berries, and insects—including stink bugs (Pentatomidae) [34,35]. Adult Northern cardinals forage on the ground as well as in shrubs and trees [36]. Adult Tufted titmice hunt for insect prey near the ground and in trees [37]. Carolina wrens are also omnivorous, but prey more so on arthropods than plant material [38]. Despite a rather small body size (approximately 20 g), analysis of Carolina wren stomach contents shows they forage on lizards, frogs, and snakes, in addition to arthropods [38]. Carolina wrens search for prey on the ground or on the trunks of trees or shrubs, and break larger prey items into smaller pieces for consumption [39]. At our study sites, all three species were common in forests near peach and pecan orchards. Furthermore, based on their foraging behavior, all three species likely encountered *H. halys* in forest habitats.

The size of *H. halys* populations in non-crop habitats fluctuates seasonally, depending on management schedules and food availability either within or surrounding orchards [6,8,9,40]. The timing of *H. halys* predation by Carolina wrens and Tufted titmice in peach and interplanted peach–pecan orchards occurred during late July or early August. In Georgia, this corresponds to the period of time after peach harvest, when *H. halys* disperse from peach orchards and into forests, that contain non-crop host plants, and likely into nearby pecan orchards as well [8]. Many host plants in forests and woodlands provide a source of food and serve as developmental hosts for *H. halys* [41,42,43]. Therefore, predation by these two species in forests occurred when populations in forests were high and there were few fruits available within peach orchards. The Northern cardinal consumed *H. halys* during the end of May in an interplanted orchard system. During this time of year, both peach and pecan fruits are developing and high numbers of *H. halys* were more likely to be detected within the orchards. Northern cardinals were commonly observed foraging on small insects in a variety of organic and conventionally managed row crop fields [44]. However, whether cardinals or other bird species actively forage in peach and pecan orchards is less understood. In an apple system, adult Great tits (*Parus major*) nested and foraged within organic apple orchards; however, pairs that nested in conventional orchards foraged in the surrounding non-crop habitat [45]. Thus, consumption of insect pests may depend on seasonal availability as well as management style.

Predation of *H. halys* in forests that surround orchards may occur during the time of year when adult stink bugs are overwintering. In this region, *H. halys* begin to overwinter in forests near peach and pecan orchards during the fall [8]. Adult *H. halys* emerge from overwintering sites during early spring [8]. Resident birds, such as woodpeckers and nuthatches, may glean overwintering *H. halys* from trees. Other birds, such as Brown thrashers (*Toxostoma rufum*) or Northern flickers (*Colaptes auratus*), may forage on *H. halys* from leaf litter on the ground. Thus, the present study may underestimate *H. halys* predation by birds because we focused our sampling efforts during the summer, when damage to peach and pecan fruits by *H. halys* is high [1,4]. Moreover, collection of fecal samples from additional species of birds, such as woodpeckers and nuthatches, may reveal higher incidence of *H. halys* predation, either during the peak summer or overwintering months.

Molecular analysis of avian diets in agroecosystems suggests that birds tend to consume more herbivores than beneficial insects [16,46,47]. In addition to *H. halys*, birds may contribute to the biological control of other stink bug pests such as the brown stink bug, *Euschistus servus* (Say) (Hemiptera: Pentatomidae), or additional insect pests that inhabit southeastern orchard agroecosystems. Two common pests found on peach trees include the lesser peach tree borer (*Synanthedon pictipes*) (Grote and Robinson) (Lepidoptera: Sesiidae) and the peach tree borer (*Synanthedon exitiosa*) (Say) (Lepidoptera: Sesiidae). Several pests damage pecan trees and nuts, including the pecan budmoth (*Gretchena bolliana*) (Slingerland) (Lepidoptera: Tortricidae), pecan nut casebearer (*Acrobasis nuxvorella*) (Neunzig) (Lepidoptera: Pyralidae), and hickory shuckworm (*Cydia caryana*) (Fitch) (Lepidoptera: Tortricidae). Birds may consume pecan budmoth, nut casebearer, and hickory shuckworm in pecan orchards, as many birds preferentially forage on or provision Lepidoptera to their nestlings when seasonally available [48,49,50].

The brown marmorated stink bug, *H. halys*, is a global pest, and due to its dispersal ability and high number of host plants, requires a multifaceted management strategy [51]. Here, we provide an initial report that birds consume *H. halys* in forests that surround peach and pecan orchards. In addition to birds, arthropod natural enemies, such as stink bug egg parasitoids contribute biocontrol services in habitats that surround orchards [52]. To promote natural enemies outside of forests, preserving and/or maintaining forest habitat and wildflower sources adjacent to orchards may provide refuge for both arthropod predators and parasitoids of *H. halys* as well as birds [53]. To promote insectivorous birds, placement of nest boxes, for species such as Western bluebirds (*Sialia Mexicana*), near orchards may increase insect pest control [46], or falcon nest boxes, which may reduce the presence of fruit-eating birds within orchards [54]. Future studies of bird foraging patterns in peach and pecan orchard agroecosystems should seek to identify the consumption of additional insect pests as well as *H. halys*.

## Figures and Tables

**Table 1:** Summary of bird species and total number of avian fecal samples for each species collected during 2021 and 2022 in peach and pecan orchards in Peach County, Georgia, USA.

Common Name	Scientific Name	2021	2022	Total
Blue-gray gnatcatcher	*Polioptila caerulea*	1	0	1
Blue Jay	*Cyanocitta cristata*	1	0	1
Brown Thrasher	*Toxostoma rufum*	0	1	1
Carolina Chickadee	*Poecile carolinensis*	4	1	5
Carolina Wren	*Thryothorus ludovicianus*	64	20	84
Eastern Kingbird	*Tyrannus tyrannus*	1	0	1
Eastern Towhee	*Pipilo erythrophthalmus*	21	5	26
Eastern Wood Pewee	*Contopus virens*	4	0	4
Field Sparrow	*Spizella pusilla*	24	8	32
Great Crested Flycatcher	*Myiarchus crinitus*	4	2	6
Gray Catbird	*Dumetella carolinensis*	0	1	1
Indigo Bunting	*Passerina cyanea*	4	1	5
Northern Cardinal	*Cardinalis cardinalis*	30	7	37
Northern Mockingbird	*Mimus polyglottos*	1	0	1
Pine Warbler	*Setophaga pinus*	0	9	9
Summer Tanager	*Piranga rubra*	1	4	5
Tufted Titmouse	*Baeolophus bicolor*	13	6	19
White-eyed Vireo	*Vireo griseus*	11	7	18
Wood Thrush	*Hylocichla mustelina*	1	0	1

## Data Availability

The data presented in this study are available in the article.

## Supplementary Materials

The following supporting information can be downloaded at: https://www.mdpi.com/article/10.3390/insects14070595/s1, Table S1: Positive consumption of *H. halys* by birds.
