# Supplementary material for: Low Incidence of Avian Predation on the Brown Marmorated Stink Bug, Halyomorpha halys (Hemiptera: Pentatomidae), in Southeastern Orchard Systems
Source: Insects. 2023 Jul 1;14(7):595. doi: 10.3390/insects14070595 (PMC10380626; doi:10.3390/insects14070595)
Supplement: Supplementary file 1 [file insects-14-00595-s001.zip › insects-2422815-supplementary.pdf]

Supplemental Materials Table 1: Positive incidence of *H. halys* consumption by birds. We detected consumption of the brown marmorated stink bug (*H. halys*) in four out of 257 avian fecal samples. We considered a positive incidence of predation on *H. halys* if RFUs >0.075. For each bird species the USGS aluminum band prefix and band code, RFU values, and concentration (ng/uL) are shown. RFU values and concentration were visualized on a Qiagen Qiaxcel Advanced System.

Sample	Band prefix	Band code	RFU	Concentration (ng/uL)
Tufted titmouse	2941	24633	0.17	0.25
Carolina wren	2941	24656	0.157	0.24
Carolina wren	2941	24664	0.077	0.12
Northern cardinal	2841	26539	0.151	0.22
Positive control	-	-	0.76	1.9
Positive control	-	-	1.564	2.43
